# P-120. Breaking the Barrier? Vancomycin Versus Alternative Agents for Treatment of Methicillin-Resistant Staphylococcus aureus Epidural Abscess

**DOI:** 10.1093/ofid/ofaf695.348

**Published:** 2026-01-11

**Authors:** Luqman Croal-Abrahams, Mohammad Mahdee E Sobhanie, Courtney Hebert, Ashley Lipps

**Affiliations:** The Ohio State University Wexner Medical Center, Columbus, OH; The Ohio State University Wexner Medical Center, Columbus, OH; The Ohio State University, Columbus, Ohio; The Ohio State University Wexner Medical Center, Columbus, OH

## Abstract

**Background:**

The necessity of central nervous system (CNS) penetrating antimicrobials for treatment of a spinal epidural abscess (SEA) is unknown. Vancomycin remains the mainstay of treatment for methicillin-resistant *Staphylococcus aureus* (MRSA) SEA due to its ability to permeate the CNS space, however disadvantages include its associated toxicities and need for therapeutic drug monitoring. Alternative agents may be used, but data on their outcomes are limited. This study compares clinical outcomes of vancomycin versus non-vancomycin therapies for MRSA SEA.

**Table 1:**
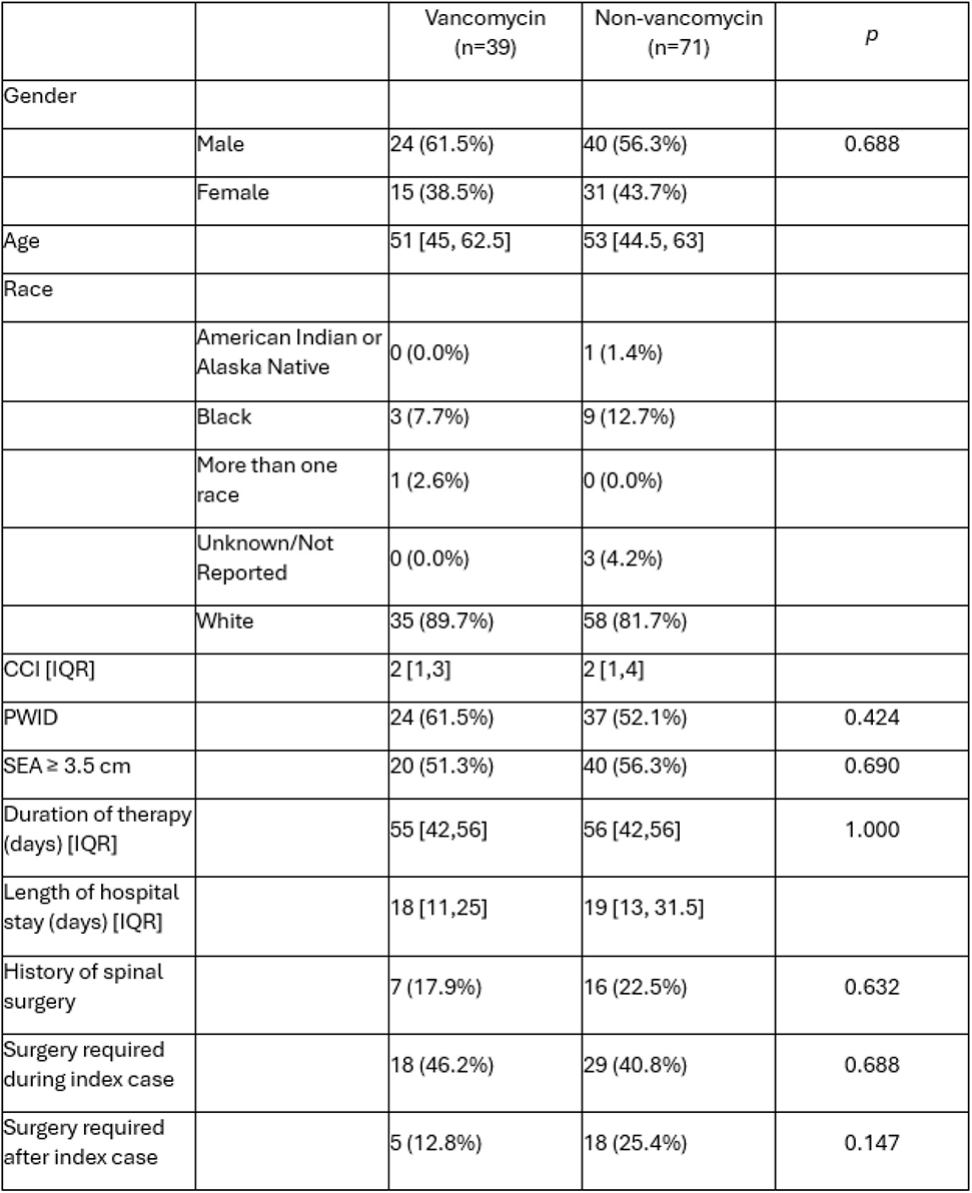
Baseline subject characteristics. CCI, Charlson Comorbidity Index; PWID, persons who inject drugs; SEA, spinal epidural abscess.

**Table 2:**
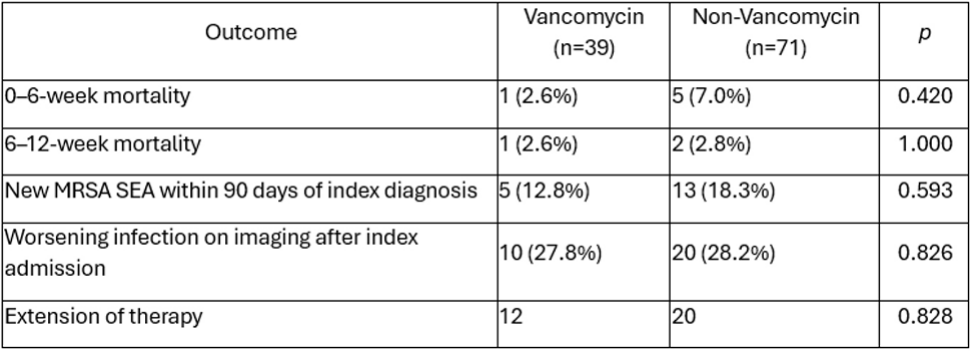
Study outcomes. MRSA, methicillin-resistant Staphylococcus aureus; SEA, spinal epidural abscess.

**Methods:**

This was a single-center, retrospective study at the Ohio State Wexner Medical Center in patients admitted from 1/1/2013 to 10/1/2024 with an ICD-9/10 code of SEA with MRSA bacteremia (MRSA-B). Subjects who were incarcerated, pregnant, miscoded, duplicated, or without MRSA-B were excluded. Subjects were assigned to the vancomycin group (VG) if they received greater than fifty percent of their treatment days with vancomycin and the remainder to the non-vancomycin group (NVG). Clinical outcomes of interests were mortality at 0-6 and 6-12 weeks from diagnosis, new diagnosis of MRSA SEA within 90 days of index diagnosis, imaging with worsening infection during treatment course, and need for therapy extension. Fisher’s exact test was used to compare the outcomes between groups.

Figure 1

**Figure:**
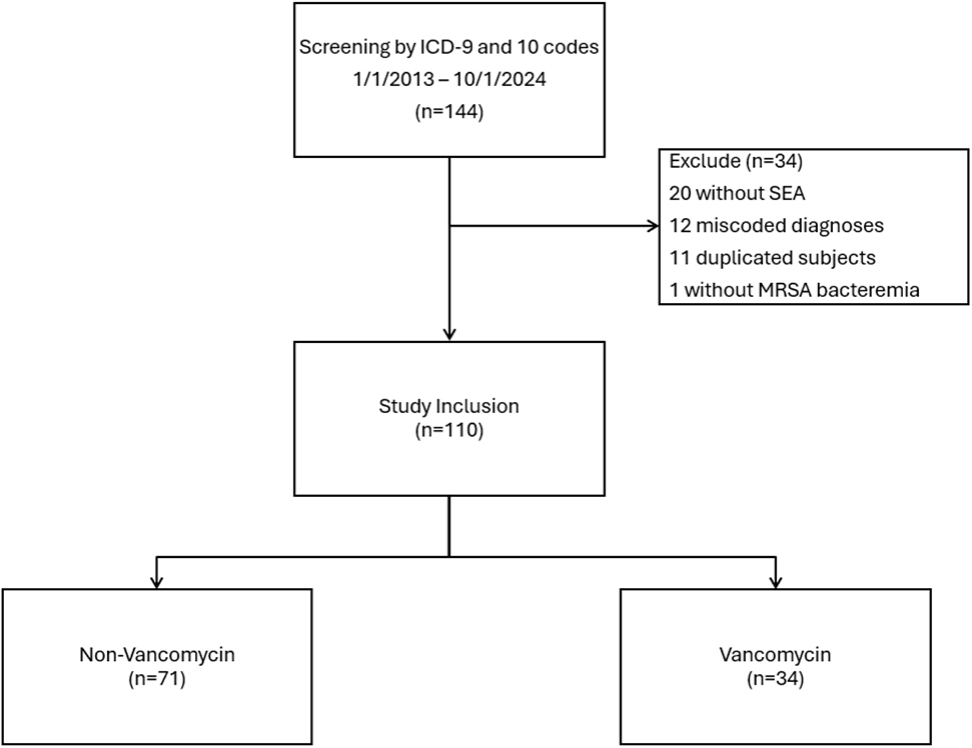

Flow diagram of participant inclusion and exclusion criteria. A total of 144 patients were screened for inclusion. Thirty-four patients were excluded. Thirty-four patients were diagnosed with MRSA SEA and treated with vancomycin, and 71 patients were diagnosed with MRSA SEA and treated with a non-vancomycin agent. MRSA, methicillin-resistant Staphylococcus aureus; SEA, spinal epidural abscess.

**Results:**

After exclusions (figure 1), 110 patients met inclusion criteria with 39 patients assigned to the VG and 71 patients in the NVG. In the NVG, the majority were treated with daptomycin monotherapy (34, 47.8%), daptomycin and ceftaroline combination therapy (19, 26.8%), or ceftaroline monotherapy (16, 22.5%). Baseline characteristics were similar between study groups (table 1). Mortality at 0-6 weeks was 2.6% in the VG and 7.0% in the NVG (p=0.42). At 6-12 weeks, mortality for these groups was 2.6% and 2.8% (p=1.00). Incidence of recurrent MRSA SEA, worsening infection on serial imaging, and extension of therapy was not different between the study groups (table 2).

**Conclusion:**

There was no significant difference in rate of mortality, recurrent MRSA SEA, worsening infection on imaging, or extension of therapy between patients in the VG versus the NVG. In this descriptive study we did not see a difference in clinical outcomes between the groups.

**Disclosures:**

All Authors: No reported disclosures

